# Supplementary material for: Real-World Data about Commonly Used Antibiotics in Long-Term Care Homes in Australia from 2016 to 2019
Source: Antibiotics (Basel). 2023 Aug 31;12(9):1393. doi: 10.3390/antibiotics12091393 (PMC10525936; doi:10.3390/antibiotics12091393)
Supplement: Supplementary file 1 [file antibiotics-12-01393-s001.zip › antibiotics-2540333-supplementary.pdf]

Supplementary File S1

Follow up time

Follow up time per resident = last cease date of any medication – first start date of any medication

Number of resident days per 1000 resident days and year

$$Numer\ of\ resident\ days\ in\ year\ 1 = \frac{\Sigma\ Follow\ up\ time\ in\ year\ 1}{1000}$$

Monthly prevalence of antibiotic use

$$Monthly\ prevalence\ of\ antbiotic\ use = \frac{\Sigma\ residents\ on\ \geq 1 \text{ antibiotic or more in that month}}{\Sigma\ number\ of\ residents\ present\ in\ LTC\ in\ that\ month}\ x\ 1000$$

Duration of antibiotic use per resident

Duration of use = last date of antibiotic episode – first date of antibiotic episode

Duration per three months per calendar year

$$Average\ number\ of\ days\ of\ antibiotic\ therapy\ per\ 100\ days\ in\ year\ 1 = \frac{\Sigma\ duration\ of\ use\ in\ year\ 1}{\Sigma\ Follow\ up\ time\ in\ year\ 1}$$

Percentage of antibiotic users per antibiotic duration of use category

5 duration levels: ≤ 5 days; 6-14 days; 15 – 30 days; 31 – 90 days; 91 – 365 days and > 365 days over the total study period. Formula given for 5 or less than 5 days of duration.

$$\%\ Duration\ of\ use\ for\ \leq 5\ days = \frac{\Sigma\ Number\ of\ antibiotic\ episodes\ with\ durations \leq 5\ days}{\Sigma\ Total\ number\ of\ antibiotic\ episodes\ in\ dataset}\ x\ 100$$
